# 

**Published:** 1887-02

**Authors:** 


Whole Number, 326. FEBRUARY, 1887. Vol. LIV. Number 2.

LIBRARY
INDEXED
S.G.O.

INDEX
MEDICUS

### THE CHICAGO MEDICAL JOURNAL AND EXAMINER

Editor: S. J. JONES, M.D., LL.D.

PUBLISHED MONTHLY BY

THE CLARK & LONGLEY COMPANY,

Under direction of

THE CHICAGO MEDICAL PRESS ASSOCIATION,

308-316 DEARBORN STREET.

